# Adaptive seamless clinical trials using early outcomes for treatment or subgroup selection: Methods, simulation model and their implementation in R

**DOI:** 10.1002/bimj.201900020

**Published:** 2020-03-02

**Authors:** Tim Friede, Nigel Stallard, Nicholas Parsons

**Affiliations:** ^1^ Department of Medical Statistics University Medical Center Göttingen Göttingen Germany; ^2^ Division of Health Sciences Warwick Medical School University of Warwick Coventry UK

**Keywords:** adaptive design, clinical trials, closed test procedure, combination test, Dunnett test

## Abstract

Adaptive seamless designs combine confirmatory testing, a domain of phase III trials, with features such as treatment or subgroup selection, typically associated with phase II trials. They promise to increase the efficiency of development programmes of new drugs, for example, in terms of sample size and/or development time. It is well acknowledged that adaptive designs are more involved from a logistical perspective and require more upfront planning, often in the form of extensive simulation studies, than conventional approaches. Here, we present a framework for adaptive treatment and subgroup selection using the same notation, which links the somewhat disparate literature on treatment selection on one side and on subgroup selection on the other. Furthermore, we introduce a flexible and efficient simulation model that serves both designs. As primary endpoints often take a long time to observe, interim analyses are frequently informed by early outcomes. Therefore, all methods presented accommodate interim analyses informed by either the primary outcome or an early outcome. The R package asd, previously developed to simulate designs with treatment selection, was extended to include subgroup selection (so‐called adaptive enrichment designs). Here, we describe the functionality of the R package asd and use it to present some worked‐up examples motivated by clinical trials in chronic obstructive pulmonary disease and oncology. The examples both illustrate various features of the R package and provide insights into the operating characteristics of adaptive seamless studies.

## 1 INTRODUCTION

There is a long history of application of sequential methods in clinical trials to allow the monitoring of accumulating data at a series of interim analyses (Jennison & Turnbull, 1999; Whitehead, 1997). Whilst most early work focussed on the aim of stopping the trial as soon as sufficient evidence has been obtained, this body of work has rapidly expanded to include the use of interim data for other design adaptations, including sample size reestimation (Friede & Kieser, 2006; Proschan, 2009). Over the past years, there has been considerable interest in using interim analyses for selection of treatments, with less effective treatments dropped from the study, or for selection of patient subgroups, with recruitment following an interim analysis limited to subgroup(s) in which a promising effect is indicated (Bauer, Bretz, Dragalin, König, & Wassmer, 2016; Pallmann et al., 2018).

A major statistical challenge in the development of such methods is the control of the type I error rate when adaptations are made on the basis of data that will also be included in the final analysis. This can be achieved by the combination test approach of Bauer and Köhne (1994), which yields flexible designs for treatment selection (see, e.g., Bauer and Kieser, 1999; Posch et al., 2005; Bretz, Schmidli, König, Racine, and Maurer, 2006) and subgroup selection (see Brannath et al., 2009; Jenkins, Stone, and Jennison, 2011; Wassmer and Dragalin, 2015).

Earlier work has assumed that the adaptations would be informed by the pre‐specified primary outcome, which is also used for hypothesis testing. From a practical perspective, however, this can be a strong limitation. In particular, in chronic diseases clinically meaningful endpoints might take some time to observe, which means that most or all patients are recruited by the time the primary outcome is observed for the first patients. This is illustrated, for example, by Chataway et al. (2011) in the context of secondary progressive multiple sclerosis. As a consequence, adaptations need to be based on early outcomes for adaptive designs to be feasible in these situations. Therefore, some adaptive seamless designs have been extended to allow the use of short‐term endpoint data for decision‐making at interim while the pre‐specified primary endpoint is used for hypothesis testing (Friede et al., 2011; Friede, Parsons, & Stallard, 2012; Jenkins et al., 2011; Kunz, Friede, Parsons, Todd, & Stallard, 2014, 2015; Stallard, 2010; Stallard, Kunz, Todd, Parsons, & Friede, 2015).

It is acknowledged that these more complex designs require intensive simulation studies in the planning to evaluate their operating characteristics (Benda, Branson, Maurer, & Friede, 2010; Friede et al., 2010). A limitation to the use of adaptive methods in practical applications is often the availability of software to enable construction and evaluation of appropriate study designs and to conduct the final analysis. A number of commercial software packages including ADDPLAN and EAST are available for this purpose. Although some R packages for group‐sequential designs including including gsDesign and gscounts and adaptive group‐sequential designs such as rpact are available from CRAN, there is still a shortage of comprehensive freely available software for adaptive seamless designs with treatment or subgroup selection, with the exception of the R package asd developed to plan clinical trials with treatment selection (Parsons et al., 2012).

Using the combination test approach, the aim of this paper is to present, to our knowledge for the first time, designs for treatment and subgroup selection in a unified notation and to present an efficient simulation model for their evaluation. By expressing the subgroup selection problem in a similar setting to that of treatment selection, the R package asd, originally developed for treatment selection, was extended to include designs of both types, that is, with subgroup or treatment selection. The methods implemented are based on the combination testing approach with at most two design stages. The designs obtained are fully flexible, controlling the type I error rate for any data‐driven adaptation including treatment or subgroup selection as well as sample size adaptation.

Although the methods we propose can be based on *p*‐values obtained from either summary statistics or individual patient data, in order to provide a general and efficient simulation model, we base this on the simulation of standardized sufficient statistics. The simulation model assumes multivariate normal distributions for the test statistics, but not for the individual observations. Therefore, the simulation model is not only widely applicable but the simulations are also fast. Furthermore, early outcomes informing the interim decisions are incorporated, since this is often important in practice as explained above. The application of the methods using the R package asd will be illustrated by clinical trials in chronic obstructive pulmonary disease (COPD) and oncology with treatment and subgroup selection, respectively.

## 2 METHODS

### 2.1 Notation and hypotheses

We consider first the setting of treatment selection designs. The study is conducted in up to J≥2 stages. In the first stage, patients are randomized between *K* experimental treatments and a control treatment. In a general setting, suppose that observations of the pre‐specified primary outcome from treatment group *k*, k=0,…,K, where k=0 corresponds to the control treatment, have a distribution depending on some parameter $\mu_{k}$, and that it is desired to test the family of null hypotheses $H_{k}:\theta_{k}=0$, k=1,…,K, where $\theta_{k}=\mu_{k}-\mu_{0}$. Let $p_{kj}$ denote a *p*‐value for the test of the null hypothesis $H_{k}$ based on the data from patients first observed in stage *j*. These data may not be available at the time of the interim analysis following stage *j*, but become available only later on in the trial (Friede et al., 2011). As we will explain in Section 2.2 below, the interim decisions need not necessarily be based on the primary outcome but could, in principle, make use of any available outcome while still maintaining control of the type I error rate.

Next we consider the setting of subgroup selection designs. Suppose that a single experimental treatment is to be compared with a control treatment but that patients can be categorized as belonging to one or more predefined subgroups. Interest is focused on the treatment effect in the full population and each subgroup, in particular in the subgroup with the largest treatment effect.

As part of the aim of this paper is to make explicit the similarities between methodology for treatment and subgroup selection designs, we will use similar notation for both settings when this does not cause confusion. Thus suppose that observations come from patients in *K* subgroups (including the full population as a special case) labeled k,k=1,…,K. Suppose that data for patients in subgroup *k* receiving treatment r,r=0,1 have a distribution depending on some parameter $\mu_{kr}$. Setting $\theta_{k}=\mu_{k1}-\mu_{k0}$, it is desired to test the family of hypotheses $H_{k}:\theta_{k}=0$, k=1,…,K. As above, $p_{kj}$ denotes a *p*‐value for the test of null hypothesis $H_{k}$ based on the data from patients with an outcome first observed in stage *j*.

### 2.2 Interim selection rules

Although in both the treatment and subgroup selection settings, the testing strategies described here control the type I error rate for any selection rule, it is good practice to specify selection rules in advance to enable calculation of operating characteristics including sample size and power. In the following, we introduce some possible examples of interim selection rules based on the primary outcome, though these could equally be applied to an early outcome as we will explain below.

One obvious way to proceed would be to select the treatment or subgroup which performs best in terms of some statistic $Z_{j,k}$ (where $Z_{j,k}$ is some estimator of $\theta_{k}$ following stage *j*). However, this might not be wise in situations where sample sizes are relatively small given the differences between the treatments or subgroups, since there is a rather high risk of picking some treatment or subgroup that is not optimal. The so‐called ε‐rule proposed by Kelly et al. (2005) selects all treatments or subgroups with statistics $Z_{j,k}$ for which $Z_{j,k}\geq \max_{i}Z_{j,i}-\varepsilon$ (assuming that larger values of $Z_{j,k}$ are better). For ε=0, this rule reduces to selecting the maximum only. For large ε, no selection takes place as all treatments or subgroups are carried forward. Otherwise varying numbers of treatments or subgroups are carried forward into the next stage. For practical applications, it is advisable to study the operating characteristics of the design for a range of values for ε. Comments on the optimal choice of ε are provided in Section 4 of Friede and Stallard (2008). The authors conclude that “selecting only the best treatment is a simple selection rule which is often optimal or close to optimal” (Friede & Stallard, 2008).

Multi‐arm studies including several doses of an experimental drug motivate another selection rule. In some indications, it is not uncommon to select not one but two doses for confirmatory testing in phase III. The COPD study discussed in more detail in Section 5 is a good example for this. A more generalized version of this rule would be to select the best $K^{★}$ out of *K* treatments or subgroups where $K^{★}$ would be specified in advance.

The selection of treatments or subgroups in interim analyses could be informed by the primary outcome or, if this is not feasible, by an early outcome. In situations where an early outcome informs an interim adaptation and the primary endpoint is used for hypothesis testing, we refer to the primary endpoint also as the final outcome to distinguish it more clearly from the early outcome. The early outcome need not necessarily fulfill all requirements of a surrogate endpoint (Burzykowski, Molenberghs, & Buyse, 2005) as weaker conditions might suffice. Chataway et al. (2011) used the phrase of a “biologically plausible” outcome that “gives some indication as to whether the mechanism of action of a test treatment is working as anticipated”. Nevertheless, for the operating characteristics of the adaptive seamless design the correlation between the early and the final outcomes on an individual patient level as well as the treatment effects on both the early and the final outcome (population level) are relevant as we will see below.

### 2.3 Error rate control via the closed testing procedure

In either the treatment selection or the subgroup selection setting, the problem has been posed in such a way that it is desired to test the null hypotheses $H_{k}:\theta_{k}=0,k=1,\ldots ,K$. It is desirable to conduct these hypotheses tests so as to control the familywise error rate in the strong sense, that is to control the probability of rejection of any true null hypothesis within this family, at some specified level, α. Strong error rate control may be achieved through a closed testing procedure in which, denoting by $H_{K}$ the intersection hypothesis $\cap_{k\in K}H_{k}$, all hypotheses $H_{K}$ for K⊆{1,…,k} are tested at nominal level α, and $H_{k}$ rejected if and only if $H_{K}$ is rejected at this level for all K∋k (Marcus, Peritz, & Gabriel, 1976).

Application of the closed testing procedure requires a test of the intersection hypothesis $H_{K}$ for each K⊆{1,…,k}. These hypotheses tests must also combine evidence from the different stages in the trial. This may be achieved through the use of a combination testing method, as described in detail in the next subsection.

### 2.4 The combination testing method and early stopping

Although the treatment or subgroup selection might be informed by an early outcome, hypothesis testing is for the primary outcome. Extending the notation introduced above, let $p_{Kj}$ denote a *p*‐value for a test of the null hypothesis $H_{K}$ based on data from patients with an outcome first observed at stage j=1,⋯,J. By construction, under $H_{K}$, $p_{Kj}∼U\left[0,1\right]$, or if a conservative test is used, $p_{Kj}$ is stochastically larger than or equal to *U*[0, 1], for all *j* and K (Brannath, Posch, & Bauer, 2002). We also assume that the conditional distribution of $p_{Kj}$ given $p_{K1},\ldots ,p_{Kj-1}$ is stochastically no smaller than a *U*[0, 1] for all $p_{K1},\ldots ,p_{Kj-1}$ for all *j*, which is also referred to as the *p‐clud* condition (Brannath et al., 2002). The condition is satisfied if the *p*‐values from different stages are independent. In practical applications, the *p‐clud* condition is only satisfied asymptotically and referred to as *asymptotically p‐clud* (Brannath et al., 2009). In the following, we assume that all *p*‐values are (at least asymptotically) *p‐clud*.

The *p*‐values from the different stages can be combined using a number of combination functions (Bauer & Köhne, 1994; Lehmacher & Wassmer, 1999) to give test statistics $C_{Kj}\left(p_{K1},\ldots ,p_{Kj}\right)$ which, under the assumptions above regarding the distributions of the *p*‐values, have known distributions under $H_{K}$ irrespective of adaptations made to the study design. These test statistics may thus be used to test hypothesis $H_{K}$ (Bauer & Kieser, 1999; Bretz et al., 2006).

Although a number of combination functions have been proposed, here we use the inverse normal combination function (Lehmacher & Wassmer, 1999), which is equivalent to the method of Cui, Hung, and Wang (1999). This gives test statistics $C_{Kj}=\sum_{j^{\prime }=1}^{j}w_{j^{\prime }}\Phi^{-1}\left(1-p_{Kj^{\prime }}\right)$ where $w_{1},\ldots ,w_{J}$ are specified in advance and the sum of their squares is equal to 1, that is, $\sum_{j=1}^{J}w_{j}^{2}=1$. Given the distributional assumptions under $H_{K}$, $C_{Kj}$ are distributed as, or are stochastically no larger than, a multivariate normal distribution with mean zero, $var\left(C_{Kj}\right)=w_{1}^{2}+\cdots +w_{j}^{2}$ and $cov\left(C_{Kj},C_{Kj^{\prime }}\right)=w_{1}^{2}+\cdots +w_{\min\{j,j^{\prime }\}}^{2}$.

Following Posch et al. (2005), we assume that hypotheses cannot be rejected once they are dropped, resulting in conservative tests. Furthermore, applying the closed testing principle outlined in Section 2.3 in an adaptive design the *p*‐value $p_{Kj}$ is replaced by $p_{K\cap I_{j}j}$ where $I_{j}$ is the set of hypotheses carried forward into stage *j* (Posch et al., 2005).

If interim analyses are used for adaptation of the design but not for stopping the trial, a final test of $H_{K}$ may be based on $C_{KJ}$. More generally, a sequential test of $H_{K}$ may be conducted based on the joint distribution of $C_{K1},\ldots ,C_{KJ}$, rejecting $H_{K}$ if $C_{Kj}\geq u_{Kj}$ for some critical values $u_{K}={\left(u_{K1},\ldots ,u_{KJ}\right)}^{T}$. To simplify the notation, $u_{K}$ and $u_{Kj}$ will generally be written as *u* and $u_{j}$ when it is clear which hypothesis is tested. The single constraint that the overall error rate should be at most α is insufficient to determine *u* uniquely. A common approach in sequential analysis is to specify the type I error to be spent at each interim analysis, and to find $u_{K1},\ldots ,u_{KJ}$ to satisfy

(1) $$pr_{H_{K}}\left(C_{Kj^{\prime }}\geq u_{Kj^{\prime }},\;\text{some}\;j^{\prime }\leq j\right)=\alpha_{j}^{*},$$

where $\alpha_{1}^{*}\leq \cdots \leq \alpha_{J}^{*}=\alpha$ are either specified in advance (Slud & Wei, 1982) or depend on the observed information in some predetermined way (Lan & DeMets, 1983). Critical values $u_{K1},\ldots ,u_{KJ}$ satisfying (1) can be found recursively with $u_{Kj}$ found directly from the joint distribution of $C_{H_{K}1},\ldots ,C_{H_{K}J}$ via a numerical search once $u_{K1},\ldots ,u_{Kj-1}$ are known. Computational details are given by, for example, Jennison and Turnbull (1999).

Construction of the sequential test statistics $C_{K1},\ldots ,C_{KJ}$ requires specification of *p*‐values $p_{K1},\ldots ,p_{KJ}$ for testing $H_{K}$. For elementary hypotheses, that is when |K|=1, $p_{Kj}$ can be obtained from a standard test, such as a *t*‐test for normally distributed data or a chi‐squared test for binary data. When |K|>1, $p_{Kj}$ should be calculated so as to allow for the multiple comparisons implicit in testing $H_{K}$. In the treatment selection setting, as comparisons are with a common control, a Dunnett test (Dunnett, 1955) may be used. In the subgroup selection setting, the simple Bonferroni procedure, Simes' procedure (Brannath et al., 2009) or the Spiessens–Debois test (Spiessens & Debois, 2010), a Dunnett‐type test with a generalized covariance structure, may be used. In each case, the level of adjustment depends on the size of K.

## 3 SIMULATION MODEL

As eluded to in Section 1, simulations are often required in the planning phase of a study to choose design options such as sample sizes or interim selection rules. Here, we propose a simulation model that is efficient in the sense that population statistics are generated rather than individual patient data. Therefore, computation times do not increase with larger sample sizes.

In a wide variety of settings, it is possible to obtain statistics that are, at least asymptotically, normally distributed with known variance. When a series of interim analyses is conducted, these statistics follow a multivariate normal distribution with non‐zero correlations, since data obtained at earlier stages are also used at later ones. In the setting of treatment or subgroup selection, these multivariate normal distributions may be extended to give the joint distribution of statistics corresponding to different treatment comparisons or for treatment effects in different subgroups. To be clear, these statistics are not actually necessarily the test statistics used to test the hypotheses but are rather some estimators of $\theta_{k}$. Similarly, the distributional forms assumed in the simulations need not be used as the basis of hypotheses tests, since these, as described in Section 2.4, can use the combination testing approach.

When considering adaptations to the trial design based on the observation of short‐term endpoint data, it is helpful to further extend the simulation models to include test statistics calculated based on different endpoints. The distributions of the resulting test statistics are described briefly in this section. Additional detail is given in Appendix.

### 3.1 Treatment selection

Considering treatment selection designs, let ${\hat{\theta }}_{kj}$ denote the estimate of the treatment effect, $\theta_{k}$, for treatment *k* based on the data available at the *j*th interim analysis, and $I_{kj}^{-1}$ denote the variance of this estimate. As these estimates are often, at least asymptotically normally distributed, our model will be based on an assumption of this distributional form. Assume that $I_{kj}$ does not depend on *k*, and so may be denoted by $I_{j}$, and that the correlation between different treatment comparisons with a common control group is 1/(1+λ), as will often be the case if we have 1:λ randomization. Setting $S_{kj}={\hat{\theta }}_{kj}I_{kj}$, we then have

$${\left(S_{11},\ldots ,S_{K1},\ldots ,S_{1J},\ldots ,S_{KJ}\right)}^{\prime }∼N\left(I⊗\theta ,\Sigma^{\left(GS\right)}\left(I\right)⊗\Sigma_{K}^{\left(CS\right)}\left(\frac{1}{1+\lambda }\right)\right),$$

where *I* and θ denote, respectively, the vectors ${\left(I_{1},\ldots ,I_{J}\right)}^{\prime }$ and ${\left(\theta_{1},\ldots ,\theta_{K}\right)}^{\prime }$ and

$$\Sigma_{K}^{\left(CS\right)}\left(r\right)=\left(\begin{array}{cccc} 1 & r & \cdots & r\\ r & 1 & \ddots & \vdots \\ \vdots & \ddots & \ddots & r\\ r & \cdots & r & 1 \end{array}\right)$$

and

$$\Sigma^{\left(GS\right)}{\left(I\right)}^{\left(1\right)}=\left(\begin{array}{cccc} I_{1}^{\left(1\right)} & I_{1}^{\left(1\right)} & \cdots & I_{1}^{\left(1\right)}\\ I_{1}^{\left(1\right)} & I_{2}^{\left(1\right)} & \cdots & I_{2}^{\left(1\right)}\\ \vdots & \vdots & \ddots & \vdots \\ I_{1}^{\left(1\right)} & I_{2}^{\left(1\right)} & \cdots & I_{J}^{\left(1\right)} \end{array}\right)$$

denote variance matrices of the K×K complex symmetric form and of that obtained in the usual group‐sequential setting.

### 3.2 Subgroup selection

In the subgroup selection setting, let ${\hat{\theta }}_{kj}$ denote the estimate of the treatment effect, $\theta_{k}$, for subgroup *k* based on the data available at the *j*th interim analysis. Again, assume that these estimates are normally distributed, let $I_{kj}^{-1}$ denote the variance of this estimate and assume that $I_{kj}$ does not depend on *k*, and so may be denoted by $I_{j}$. In the simplest case, in which K=2 and subgroup 2 is the whole population and subgroup 1 a proportion of size τ, setting $S_{kj}={\hat{\theta }}_{kj}I_{kj}$, we have

$${\left(S_{11},S_{21},\ldots ,S_{1J},S_{2J}\right)}^{\prime }∼N\left(I⊗\theta ,\Sigma^{\left(GS\right)}\left(I\right)⊗\Sigma^{\left(GS\right)}\left(\begin{array}{c} \tau \\ 1 \end{array}\right)\right).$$

Extensions to the case when a short‐term endpoint is also considered, and to more complex subgroup selection settings are discussed in the Appendix.

## 4 SOFTWARE IMPLEMENTATION IN R

### 4.1 R package asd

The simulation models for two‐stage treatment selection and subgroup selection designs, described in Section 3, can be implemented in the R (R Development Core Team, 2009) package asd (Parsons et al., 2012), which is available from the Comprehensive R Archive Network (CRAN) at http://cran.r‐project.org/package=asd). This package comprises a number of functions that allow the properties of seamless phase II/III clinical trial designs, potentially using early outcomes, for treatment or subgroup selection to be explored and evaluated prior to a study commencing. An earlier version of this package, without the extension to subgroup designs, enhanced options for a wider range of outcome measures or more complete output model description was described previously by Parsons et al. (2012). The general structure of the code comprises a set of base functions that implement lower level tasks such as hypothesis testing, treatment selection, and closed testing procedures. The base functions, which have been described previously in the setting of treatment selection designs (Parsons et al., 2012), have been modified in the latest version of the asd package to work with both subgroup and treatment selection designs. The higher level user facing function asd.sim has been replaced by two new functions treatsel.sim and subpop.sim that are called directly by the user to implement hypothesis testing and simulations. The more general functions, gtreatsel.sim and gsubpop.sim can in principle be called directly by the user, although due to the more complex input structure this is generally not recommended.

### 4.2 Function subpop.sim

Friede et al. (2012) extended the previously described combination test (CT) approaches, for co‐primary analyses in a single pre‐defined subgroup and the full population, using the methods proposed by Spiessens and Debois (2010) to control the familywise error rate (FWER) in the subgroup and the full population, and also proposed a novel method to obtain a critical value for the definitive test using a conditional error function (CEF) approach; full details of these methods are given by Friede et al. (2012). The function subpop.sim implements all the methods for subgroup selection in adaptive clinical trials reported by Friede et al. (2012) and subsequent correspondence (Friede, Parsons, & Stallard, 2013). The authors described and explored the performance of a number of methods in the setting described here, distinguishing between two distinct approaches to control the FWER, a CT method (Brannath et al., 2009; Jenkins et al., 2011) and a CEF method.

#### 4.2.1 Input arguments

An overview and brief description of the input arguments available for subpop.sim is shown in Table 1. The CT methodology described by Friede et al. (2012) can be implemented in subpop.sim using either the Spiessens and Debois (2010) (SD), Simes or Bonferroni testing procedures to control the FWER. These and the CEF approach can be selected using the method argument to subpop.sim. The following options are available: (i) CT‐SD (method="CT-SD"), (ii) CT‐Simes (method="CT-Simes"), (iii) CT‐Bonferroni (method="CT-Bonferroni"), and (iv) CEF (method ="CEF").

**TABLE 1:** Brief description and available input arguments to R functions subpop.sim and treatsel.sim

		Implementation	
Argument	Description	Treatment selection (treatsel.sim)	Subgroup selection (subpop.sim)
n	Sample sizes for each treatment group at stage 1 (interim) and stage 2 (final) analyses	List of sample sizes; for example, 32 in each group in stage 1 and 64 in stage 2, list(stage1=32, stage2=64)	An additional list option can be used to increase (enrich) the sample size in stage 2; for example, enrich sample size to 128 in stage 2, list(stage1=32, enrich=128, stage2=64)
effect	Effect sizes for early and final outcomes	List of effect sizes for the control (first) and each treatment group; for example, for control (0) and two effects of size 0.1 and 0.2 for early and 0.2 and 0.3 for final outcome, list(early=c(0,0.1,0.2), final=c(0,0.2,0.3))	The first element of each vector is the effect size in the subgroup and the second is the effect size in the full population; for example, for an effect size of 0.4 in subgroup and 0.2 in the full population for both early and final outcomes list(early=c(0.4,0.2), final=c(0.4,0.2)). An optional argument can be included to set the effect size for the control group; for example, default is zero, control=list(early=0, final=NULL)
outcome	Outcome type for early and final outcomes	List of outcome types, options for normal (N), time‐to‐event (T), and binary (B) are currently available; for example, normal for early and final outcomes list(early="N", final="N").	
nsim	Number of simulations	An integer $<1\times 10^{7}$	
sprev	Subgroup prevalence		The prevalence of the subgroup in the main population; 0<sprev<1. Subgroup prevalence can be either fixed or allowed to vary at each simulation; default is sprev.fixed=TRUE
corr	Correlation between outcomes	Correlation between early and final outcomes; −1<corr<1	
seed	Seed number	Seed number to ensure repeatability of simulations	
select	Method for treatment selection	Seven available options (see Section 4.3.1), with default, select=0, to select all treatments. For select=4, the epsilon rule, can be used to allow more flexibility, with default epsilon=1 and for select=6, all treatments greater than a threshold can be selected, with default thresh=1	Two options are available. The default threshold selection rule (select="thresh"), for which limits must be set; for example, selim=c(-1,1). A futility selection rule (select="futility"; See Section 5.2) is also available, for which limits must be set; selim=c(0,0).
ptest	Treatments for counts of the number of rejections	A vector of valid treatment numbers for treatment specific counts of rejections; for example, for four treatments, ptest=c(1), ptest=c(1, 2) or ptest=c(1,2,3,4) are all valid options	
method	Methodology used for simulations; and for subpop.sim either	Either method="invnorm" or "fisher" to select inverse normal or Fisher combination test	Select method="CT-SD", "CT-Simes", "CT-Bonferroni" or "CEF" (see Section 4.2.1)
fu	Follow‐up options	Subjects in the dropped treatment groups can followed‐up (fu=TRUE), with default fu=FALSE (see Section 4.3.1.)	
weight	Stage 1 weight	Stage 1 weight be defined with this option (0 ⩽ weight ⩽1); default weight=NULL	
level	Test level	Test level must be set (0≤level≤1); default level=0.025	
file	File name for output	If unset will default to R console. For example, set file="output.txt", direct output to file in R working directory (getwd())	

The syntax providing the group sample sizes, for stages 1 and 2, for a putative trial design and effect sizes for early and final outcomes is consistent across the available outcome types and comprises a list of the selected options. For instance, a design where the required sample size per treatment arm is 100 for stage 1 and 200 for stage 2 would be implemented using the following expression, n=list(stage1= 100,stage2=200). The assumption, in the current version of asd, is that the sample size in the control arm of the study is the same as in the treatment arm. The default setting is that if the subgroup only is selected at the interim analysis at stage 1, then the subgroup prevalence remains the same after stage 1. If an increase in the sample size in the subgroup was planned, if this group only was selected (enrichment), then this can be implemented by adding an additional item to the list to indicate this. For instance, if we wanted a sample size of 200 in the subgroup in this setting, irrespective of the subgroup prevalence, then we would modify the previous argument to the following n=list(stage1=100,enrich=200,stage2=200).

Effect sizes for early and final outcomes are also given as a list using expressions of the following structure, where the first element of each vector is the effect size in the subgroup and the second element is the effect size in the full population. Setting effect=list(early=c(0.3,0.1),final=c(0.3,0.1)) specifies an effect size of 0.3 in the subgroup and 0.1 in the full population; the effect size in the control group is set by default to be zero. The default setting is for normal outcomes for both early and final outcomes, outcome=list(early="N",final="N"), and the effects are interpreted given these options. The available options for outcome types are normal (N), time‐to‐event (T), and binary (B), and all combinations of these are allowed for early and final outcome measures. Generally, it is assumed that higher means (N) and lower event rates (B or T) are better. A detailed description of these options is left for the following section describing function treatsel.sim; the options described there are analogous to those available for subpop.sim. In the simpler setting where group selection is based purely on the final outcome, this can be implemented by setting the effect sizes for the early and final outcomes to be equal and the correlation between the early and final outcomes to one (i.e., corr=1).

The subgroup prevalence is set by a single argument, namely (sprev). For instance, sprev=0.5 indicates that the subgroup comprises half of the full population. The function subpop.sim randomly generates test statistics (with a seed number set using seed) and accumulates results from usually a large number of simulations that must be set using the nsim option (default setting, nsim=1000). The prevalence can be either fixed at the set value (sprev.fixed=TRUE) or allowed to vary (sprev.fixed=FALSE) using a single realization of the binomial random variate generation function rbinom, at the set values for the sample size and subgroup prevalence, at each simulation.

Subgroup selection at interim is implemented using the so‐called threshold selection rule (Friede & Stallard, 2008; Friede et al., 2012) (select="thresh"). If for the difference Δ of the test statistics for the full population and the subgroup holds $\Delta \leq l_{1}$, then the subgroup only is tested at the end of stage 2. If $\Delta >l_{2}$ the full population only is tested. Otherwise both subgroup and full populations are tested. The thresholds $\left(l_{1},l_{2}\right)$ are set using the argument selim, which is a vector of standard deviation multiples. If, for instance, large limits are set (e.g., selim=c(-10,10)) then both subgroup and full populations will always be tested at the trial endpoint, whereas if selim=c(0,0), only the test regarding the population with the largest test statistic at interim is taken into stage 2. Intermediate values for selim between these extremes provide more flexible selection options. The weight for the CT approaches, if unset, is given by $n_{\text{stage}\;1}/\left(n_{\text{stage}\;1}+n_{\text{stage}\;2}\right)$ with $n_{\text{stage1}}$ and $n_{\text{stage2}}$ referring to the (full) population sizes at stage 1 and stage 2, respectively. The test level is set by default to 0.025 (level=0.025).

#### 4.2.2 Output

The output to subpop.sim first gives a summary of the simulation model, including expected values for the test statistics at each stage of the study. The main summary table reports the number of times that hypotheses $H_{0}^{\left\{S\right\}}$, $H_{0}^{\left\{F\right\}}$, $H_{0}^{\left\{S,F\right\}}$ were selected for testing and rejected when the subgroup (*S*), the full population (*F*), or both were tested. Output from subpop.sim is by default directed to the usual R console, but to save more detailed summaries of the simulation model, output can be directed to a file using this as an argument to the file function (e.g., file="output.txt"). Section 5 shows how subpop.sim is used in a practical setting with example data.

### 4.3 Function treatsel.sim

Function treatsel.sim replaces and generalizes the previous function asd.sim, with the name change made to make it much more explicit that the code implements only simulations for treatment selection designs for multi‐arm studies. Much of the syntax and model setup is consistent between treatsel.sim and subpop.sim.

#### 4.3.1 Input arguments

An overview and brief description of the input arguments available for treatsel.sim is shown in Table 1. One aspect of the design setup that differs considerably between treatsel.sim and subpop.sim is the coding of the treatment effects. Treatment effect sizes are given for the control group μ_0_ and the test treatment or treatments $\mu_{k}$ and as vectors for both early and final outcomes; for instance, effect=list(early=c(0,0.1,0.2,0.1), final=c(0,0.1,0.2,0.3)) indicates that there are three test treatments with effect sizes 0.1, 0.2, and 0.1 for the early outcome and 0.1, 0.2, and 0.3 for the final outcome, respectively; the control is set to 0 for both outcomes. The null hypotheses tested are $H_{k}:\theta_{k}=\mu_{k}-\mu_{0}=0$. There is no limit to the number of test treatment groups *K*. However, in practice, our experience is that the code runs slowly for designs with eight or more treatment groups, that is, K≥8. The setting of the effect sizes can be clarified further by considering the available options for the outcome types (normal N, time to event T, and binary B), set using the option outcome, with the default being to have both early and final outcomes normal (outcome=list(early="N",final="N")), with all nine combinations available. Generally, it is assumed that higher means (N) and lower event rates (B or T) are better.

For normal outcomes, the test statistics for the simulation model for the *K* test treatments, relative to the control group, are given by $\sqrt{n/2}\times \left(\mu_{k}-\mu_{0}\right)$. For time‐to‐event outcomes, effects are interpreted as minus log hazard rates, with the control μ_0_ set to zero and test statistics are given by $\sqrt{o_{k}/4}\times \left(\mu_{k}-\mu_{0}\right)$, where the expected total number of events in the control and treatment groups $o_{k}$ for treatment *k* is calculated under an assumed exponential model to be $n\times \left(1-\exp\left(-\exp\left(-\mu_{0}\right)\right)\right)+n\times \left(1-\exp\left(-\exp\left(-\mu_{k}\right)\right)\right)$. Binary effects are characterized by log odds ratios, $\sqrt{1/o_{k}+1/\left(n-o_{k}\right)+1/o_{0}+1/\left(n-o_{0}\right)}\times \left(\mu_{k}-\mu_{0}\right)$, where $\mu_{k}$ is minus the log odds of the event and the observed number of events in treatment group *k* is $o_{k}=n\times 1/\left(1+\exp\left(\mu_{k}\right)\right)$. Some care must be taken when setting‐up the simulation model, as clearly the interpretation of the effect argument to treatsel.sim is dependent on the options selected for outcome.

The method argument to treatsel.sim allows either the inverse normal (invnorm) or Fisher's (fisher) combination test and the logical follow‐up argument (fu) determines whether (i) patients in the dropped treatment groups are removed from the trial and unknown test statistics in the dropped treatments are set to −∞ at stage 2 (fu=FALSE) or (ii) patients are kept in the trial and followed‐up to the final outcome, in the same manner as the patients recruited in stage 1 in the selected treatment groups (fu=TRUE); Friede et al. (2011) called option (i), the default setting, discontinued follow‐up and option (ii) complete follow‐up. Seven treatment selection rules based on stage 1 test statistics are available in treatsel.sim, and are chosen with the select argument; (i) select all treatments (select=0), (ii) select the maximum (select=1), (iii) select the maximum two (select=2), (iv) select the maximum three (select=3), (v) flexible treatment selection using the ε‐rule (Friede & Stallard, 2008; Friede et al., 2011), with additional argument (epsilon) (select=4), (vi) randomly select a single treatment (select=5) or (vii) select all treatments greater than a threshold, with the additional argument (thresh) (select=6).

The only additional argument available for treatsel.sim, that has not been covered in the section describing subpop.sim, is ptest. This is a vector of valid treatment numbers for determining specific counts for the number of simulations that reject the null hypothesis; for instance, for three test treatments and ptest=c(1,3), treatsel.sim will count and report the number of rejections of one or both hypotheses for testing treatments 1 and 3 against the control, in addition to the number of rejections of each of the elementary hypotheses.

#### 4.3.2 Output

The output from treatsel.sim first gives a summary of the simulation model, including expected values for the test statistics at each stage of the study. The main summary tables report (i) the number of treatments selected at stage 1, (ii) treatment selection at stage 1, that is, how often each treatment was selected, (iii) counts of hypotheses rejected at study endpoint, for each of the elementary hypotheses ($H_{0}^{\left\{1\right\}},H_{0}^{\left\{2\right\}},\ldots ,H_{0}^{\left\{K\right\}}$), and (iv) the number of times that one or more than one of the hypotheses identified in ptest are rejected. Section 5 shows how treatsel.sim is used in a practical setting with example data.

## 5 EXAMPLES

In this section, we illustrate the methods described above by two example studies using the R package asd. The first is a multi‐arm randomized controlled trial in COPD with treatment selection; which will be considered in Section 5.1. As a second example, we consider trials in oncology with time‐to‐event outcomes and subgroup selection; this will be considered in Section 5.2.

### 5.1 Clinical trial in COPD with treatment selection

Barnes et al. (2011) and Donohue et al. (2010) report a seamless adaptive design with dose selection, which was also discussed elsewhere (Cuffe, Lawrence, Stone, & Vandemeulebroecke, 2014). Patients were randomized to four doses of indacaterol (75μg, 150μg, 300μg and 600μg), active controls, and placebo control. For the purpose of illustration, we ignore the active controls in the following and use the observed results to illustrate the design process. The primary outcome was the percentage of days of poor control over 26 weeks. As recruitment for the entire study took only 6 months, the interim treatment selection could not be based on the primary endpoint. Trough forced expiratory volume in 1 s (FEV1) at 15 days was identified as a suitable early outcome to inform the interim analysis. From Figure 1 of Barnes et al. (2011), difference in trough FEV1 compared to placebo at 15 days is 150 ml, 180 ml, 210 ml, and 200 ml for the indacaterol doses 75μg, 150μg, 300μg, and 600μg, respectively. Reported 95% confidence intervals suggest two standard errors of treatment difference is approximately 60 ml, so assuming equal stage 1 sample sizes $n_{1}=110$, then the standard deviation of the measurements is approximately 220 ml. Standardized effect sizes are thus approximately 0.68, 0.82, 0.95, and 0.91 for the indacaterol doses 75μg, 150μg, 300μg, and 600μg, respectively. Regarding the final outcome days of poor control (%) over 26 weeks, we gather from the report of stage 2 results at http://clinicaltrials.gov/show/NCT00463567 that the placebo rate was 35%. Let us assume rates of 31%, 30%, 28%, and 29% for the four doses of indacaterol (75μg, 150μg, 300μg and 600μg). Based on reported standard errors, we estimate the standard deviation to be approximately 30%. Hence, the standardized effect sizes are approximately 0.13 (75μg), 0.17 (150μg), 0.23 (300μg), and 0.20 (600μg). The approximate sample sizes per arm were 100 patients in stage 1 and 300 patients in stage 2. Since the aim was to select two doses of indacaterol at the interim analysis to take into stage 2, we consider an overall sample size for the two stage of 5×100+3×300=1,400 patients. Furthermore, a moderate positive correlation between early and final outcomes of 0.4 is assumed.

**FIGURE 1:**
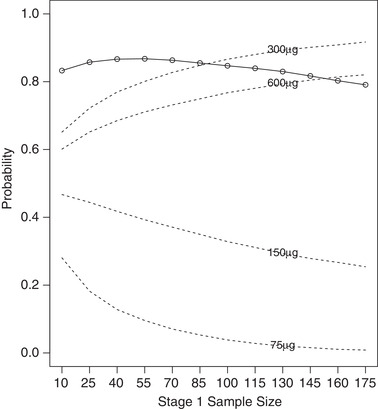
Probability of rejection of at least one elementary hypothesis (○; solid line), and selection probabilities (dashed lines) for stage 1 sample sizes in range 10–175 per group

In the following, we will consider three settings: (i) continuous early and final outcomes and selecting always two doses for the second stage, as in the original study; (ii) continuous early and final outcomes, as in the original study, but for the purpose of illustration, we vary the selection rule using the threshold rule to allow varying numbers of doses being taken forward for confirmatory testing in the second stage; (iii) as final outcomes are non‐normal and early and final outcomes are on different scales, we assume in another setting that the final is binary and not continuous.

#### 5.1.1 Continuous early and final outcomes: Selecting always two doses for the second stage

As an example, we consider a fixed total sample size of 1,400 patients, which we can allocate between the two stages, with either more or less resources for each stage. We consider the following combinations of $n_{1}=10,25,\cdots ,175$ and $5\times n_{1}+3\times n_{2}=\text{1,400}$. Each one of these sample size options can be tested using the following implementation of the treatsel.sim function for the setting with 100 patients per arm in stage 1 and 300 patients per arm in stage 2:


treatsel.sim(n=list(stage1=100,stage2=300), effect=list(early=c(0,0.68,0.82,0.95,0.91),

 final=c(0,0.13,0.17,0.23,0.20)), outcome=list(early="N",final="N"),

 nsim=10000,corr=0.4,seed=145514,select=2, level=0.025,ptest=c(3,4))





This code sets the sample sizes for each stage, and provides the effect estimates as described above, for a normal early outcome (“N”) and a normal (“N”) final outcome. The correlation is set to 0.4, and the number of simulations to 10,000. The select=2 option implements the rule that chooses the two treatments with the largest test statistics an interim. The test level is set to 0.025 and the ptest options allows us to count rejections for either or both of the doses 300μg and 600μg. Results from running this code are as follows (omitting the parts describing the setup):


simulation of test statistics:

expectation early = 4.8 5.8 6.7 6.4

expectation final stage 1 = 0.9 1.2 1.6 1.4 and stage 2 = 1.6 2.1 2.8 2.4

weights: stage 1 = 0.5 and stage 2 = 0.87



number of treatments selected at stage 1:

 n %

 1 0 0.00

 2 10000 100.00

 3 0 0.00

 4 0 0.00

Total 10000 100.00



treatment selection at stage 1:

 n %

 1 383 3.83

 2 3282 32.82

 3 8661 86.61

 4 7674 76.74



hypothesis rejection at study endpoint:

 n %

 H1 183 1.83

 H2 2067 20.67

 H3 7206 72.06

 H4 5541 55.41



reject H3 and/or H4 = 8469 : 84.69%





The first part of the output provides a summary of the model setup, and the second part values of the test statistics used in the simulations. The squared weights are calculated, in this case, as 100/400 and 300/400. The results are summarized in the three lower tables. The first indicates that two treatments were always selected at stage 1, the second gives the number of simulations in which each treatment was selected and the third gives the number of simulations in which the elementary hypotheses were rejected. The final statement gives the number of simulations in which at least one of the treatments picked using the ptest options were rejected. Assigning the output for this function to an object that we for the sake of illustration call simply output, then the summaries described here can be accessed directly, for instance, for plotting data or other analysis, using the syntax output$count.total, output$select.total, output$reject.total, and output$sim.reject.

The results of the simulations suggest that given the large treatment effects on the early outcome (trough FEV1 at 15 days), and the modest effects on the final outcome (days of poor control (%) over 26 weeks), the greatest power would have been achieved by using a sample size of around 50 per group in stage 1 (see Figure 1).

#### 5.1.2 Continuous early and final outcomes: Selecting varying numbers of doses for the second stage

As an alternative to always selecting the best two performing treatments at interim, we now consider a threshold rule, where all treatments with test statistics at interim analysis above a fixed threshold are taken into stage 2. If no treatments reach the threshold, the study is stopped for futility. Simulations are implemented using the same effect sizes as in setting 1, a stage 1 sample size of 40, and a stage 2 sample size of 400 in the following code for a threshold of 3:


treatsel.sim(n=list(stage1=40,stage2=400),

 effect=list(early=c(0,0.68,0.82,0.95,0.91),

 final= c(0,0.13,0.17,0.23,0.20)),

 outcome=list(early="N",final="N"),

 nsim=10000,corr=0.4,seed=145514,select=6,

 thresh=3,level=0.025,ptest=c(3,4))





The select=6 option implements the threshold rule, with the fixed early outcome test statistic threshold set using the thresh option. Results from running this code are as follows (omitting the parts describing the setup):


simulation of test statistics:

expectation early = 3 3.7 4.2 4.1

expectation final stage 1 = 0.6 0.8 1 0.9 and stage 2 = 1.8 2.4 3.3 2.8

weights: stage 1 = 0.3 and stage 2 = 0.95



number of treatments selected at stage 1:

 n %

 1 800 8.00

 2 1634 16.34

 3 3098 30.98

 4 4175 41.75

Total 9707 97.07



treatment selection at stage 1:

 n %

 1 5083 50.83

 2 7469 74.69

 3 8914 89.14

 4 8596 85.96



hypothesis rejection at study endpoint:

 n %

 H1 2480 24.80

 H2 4882 48.82

 H3 7769 77.69

 H4 6642 66.42



reject H3 and/or H4 = 8600 : 86%





**FIGURE 2:**
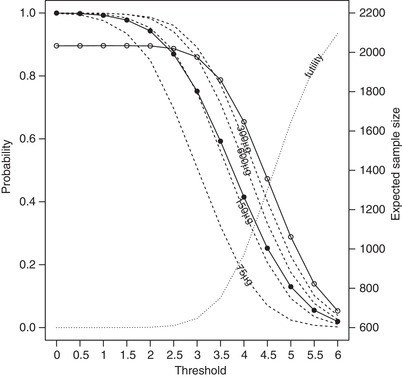
Probability of rejection of at least one elementary hypothesis (○; solid line), futility stopping (dotted line), selection probabilities (dashed lines), and expected total sample size (•; solid line) for thresholds on a standardized z‐scale in the range 0–6

Although the probability for futility stopping is not given explicitly, it can be easily derived. The considered design was stopped for futility only 3% (=100%− 97.07%) of the time; with all four experimental treatments being taken into stage 2 more than 41% of the time. Running the above code for thresholds in the range 0 to 6 (at intervals of a half) and extracting output for each option gives the results summarized in Figure 2.

The expected overall sample sizes for the scenarios in Figure 2, based on the simulated number of treatments selected at stage 1 and the fixed stage 1 and stage 2 sample sizes of 40 and 400, are as follows; 2199.5, 2197.3, 2188.1, 2164.3, 2109.0, 1991.2, 1802.5, 1548.2, 1264.0, 1004.2, 807.9, 688.2, and 631.0. The power drops off rapidly as the threshold increases from 3 to 5, as futility stopping increases from 3% to 65%. When, on average, two test treatments are taken into stage 2, that is when the fixed threshold is somewhere between 3.5 and 4 (overall sample size between 1548.2 and 1264.0), the power is lower (between 78.7% and 65.4%) than the analogous setting in Figure 1 (86.6%). The early outcome effect sizes and distributions are such that a fixed threshold that on average picks two treatments at interim, also stops for futility so often that it reduces the power considerably compared to a design that always takes exactly two treatments.

#### 5.1.3 Continuous early and binary final outcome

The implementation described here has focused on normal outcome measures for both early and final outcomes. However, if one or other outcome were binary, the changes necessary to implement this new scenario are relatively straightforward. For instance, instead of using days of poor control over 26 weeks as the final outcome measure, we might use a threshold based on this outcome. If this was the case, then success or failure of the treatment could be determined for each study participant, based on some a priori threshold for the number of days that one might expect to maintain control over the 26 week period. For the sake of example, let us consider the failure rate to be 50% in the control group, and 45% (75μg), 45% (150μg), 40% (300μg), and 40% (600μg), at each dose, respectively. Given that every other aspect of the design is the same as the first setting in the COPD example, then this design can be implemented using the following code.


treatsel.sim(n=list(stage1=100,stage2=300), effect=list(early=c(0,0.68,0.82,0.95,0.91),

 final=c(0.50,0.45,0.45,0.40,0.40)), outcome=list(early="N",final="B"),

 nsim=10000,corr=0.4,seed=145514,select=2, level=0.025,ptest=c(3,4))





This gives an overall rejection probability of 76.99%, by changing the outcome argument for the treatsel.sim function of Section 5.1.2 to list(early="N",final="B") and the vector of final effect sizes to c(0.50,0.45,0.45,0.40,0.40).

### 5.2 Clinical trials in oncology with subgroup selection

Jenkins et al. (2011) suggested designs for adaptive seamless phase II/III designs for oncology trials using correlated survival endpoints. Designs of this type can be implemented relatively straightforwardly using the subpop.sim function. Here, we explore some design properties for a typical scenario from amongst the many that Jenkins et al. (2011) explored. We assume early and final time‐to‐event outcomes with a hazard ratio of 0.6 in the subgroup and 0.9 in the full population for both, and a correlation between endpoints of 0.5; in the setting of an oncology trial, the endpoints might be progression free and overall survival. We set the stage 1 sample size to 100 patients per arm and the stage 2 sample size to 300 patients per arm, if we progress in the full population, and to 200 patients per arm if we progress in the subgroup only. The subgroup prevalence is fixed at 0.3. Using the futility rule for selection at interim, with limits for the subgroup and full population both set to 0, this scenario can be implemented using the following code:


subpop.sim(n=list(stage1=100,enrich=200,stage2=300),

 effect=list(early=c(0.6,0.9),final=c(0.6,0.9)),

 sprev=0.3,outcome=list(early="T",final="T"),

 nsim=10000,corr=0.5,seed=1234,select="futility",

 selim=c(0,0),level=0.025,method="CT-SD")





The method="CT-SD" option implements the combination test method with Spiessens and Debois testing procedure. Given test statistics at interim of *S*_1_ and *S*_2_ for the subgroup and the full population, respectively, and selection rule limits $\left(l_{1},l_{2}\right)$, the futility rule implements the following options: (i) continue with a co‐primary analysis if $S_{1}<l_{1}$ and $S_{2}<l_{2}$, (ii) continue in the subgroup alone if $S_{1}<l_{1}$ and $S_{2}>=l_{2}$, (iii) continue in the full population alone if $S_{1}>=l_{1}$ and $S_{2}<l_{2}$, and (iv) stop for futility if $S_{1}\geq l_{1}$ and $S_{2}\geq l_{2}$. Results from running this code are as follows (omitting the parts describing the setup):


simulation of test statistics:

expectation early: sub-pop = -1.46 : full-pop = -0.58

expectation final stage 1: sub-pop = -1.46 : full-pop = -0.58

expectation final stage 2: sub-pop only = -3.76 : full-pop only = -1.01

expectation final stage 2, both groups selected: sub-pop = -2.52 : full-pop = -1.01

weights: stage 1 = 0.5 and stage 2 = 0.87



hypotheses rejected and group selection options at stage 1 (n):

 Hs Hf Hs+Hf Hs+f n n%

sub 2225 0 0 2225 2309 23.09

full 0 48 0 54 227 2.27

both 5370 1658 1636 5407 6987 69.87

total 7595 1706 1636 7686 9523 -

% 75.95 17.06 16.36 76.86 95.23 -

reject Hs and/or Hf = 76.65%





The output reports that in 75.95%, 17.06%, and 16.36% of the simulations, the null hypothesis was rejected in the subgroup, full population and both, respectively. The final two columns give a breakdown of the selections made at the interim analysis; 23.09% of the simulations were continued in the subgroup only, 2.27% in the full population only, 69.87% in both, and 4.77% were stopped for futility. A concise summary of this table can be obtained for further analysis, by assigning to an output object and accessing the results using the syntax output$results.

It is informative in understanding the futility rule to run the above code for a grid of futility rule limits in the range 0 to −3; the results of this for each option are summarized in Table 2, where the notation $l_{S}$ and $l_{F}$ indicate the limits for the subgroup and full population, respectively.

**TABLE 2:** Selection probabilities, probability of futility stopping and power (probability of rejecting at least one elementary null hypothesis) for a range of values of $l_{S}$ and $I_{F}$

		Selection (%)				
$l_{F}$	$l_{S}$	Subgroup	Full	Both	Futility (%)	Power (%)
0	0	23.1	2.3	69.9	4.8	76.7
0	−1	11.4	16.2	55.8	16.7	58.8
0	−2	2.3	45.1	26.5	26.1	34.2
0	−3	0.1	66.0	6.0	27.9	20.7
−1	0	60.0	0.4	32.3	7.3	83.9
−1	−1	37.4	4.0	29.7	29.0	61.4
−1	−2	12.3	16.5	16.9	54.2	30.3
−1	−3	1.5	28.4	4.8	65.4	13.8
−2	0	84.9	0.0	7.4	7.7	88.6
−2	−1	60.1	0.3	7.2	32.4	65.0
−2	−2	24.1	2.4	5.6	68.0	29.2
−2	−3	4.4	5.3	2.1	88.2	8.0
−3	0	91.6	0.0	0.7	7.7	89.7
−3	−1	66.7	0.0	0.7	32.6	66.0
−3	−2	28.6	0.1	0.6	70.7	28.8
−3	−3	5.6	0.4	0.3	93.7	6.1

From Table 2, it is clear that as $l_{S}$ becomes more negative, the subgroup is selected progressively less often and similarly as $l_{F}$ becomes more negative, it is selected progressively more often. The balance between the two limits determines overall power in this setting. With a strong effect in the subgroup and a much weaker effect in the full population, the best strategy is to always, unless stopping for futility, test in the subgroup at the final analysis. For the grid of values tested here, this is best achieved when $l_{F}=-3$ and $l_{S}=0$.

## 6 DISCUSSION

Adaptive seamless designs are recognized as a tool to increase the efficiency of clinical development programs by combining features of learning and confirming in a single trial, while traditional development programs would have investigated these in separate trials. However, their implementation is more involved than traditional designs (see, e.g., Quinlan and Krams (2006) for a discussion). One aspect is the planning which is more complex, often requiring extensive Monte Carlo simulations (Benda et al., 2010; Friede et al., 2010). Here, we presented a unified framework for adaptive seamless designs with treatment or subgroup selection. Furthermore, we developed a flexible and yet efficient simulation model. This, as all other methods discussed, can accommodate interim selection informed by an early outcome rather than the final one. Furthermore, we demonstrate how the R package asd, freely available from CRAN, can be used to evaluate and compare operating characteristics of various designs.

Here, we employed the combination test approach to achieve type I error rate control. For treatment selection, alternative approaches build on the work of Thall, Simon, and Ellenberg (1988, 1989) using the group‐sequential method, but require that a single treatment continues along with a control beyond the first stage (Stallard & Todd, 2003) or that the number of treatments at each stage is specified in advance (Stallard & Friede, 2008). Methods based on the combination test approach are more flexible (see, e.g., Bauer and Kieser (1999), Posch et al. (2005), and Bretz et al. (2006)), but may be less powerful in some settings (Friede & Stallard, 2008). Magirr, Jaki, and Whitehead (2012) proposed a group‐sequential method that does allow completely flexible treatment selection, though this may be at the cost of conservatism, that is, decreased type I error rate below the nominal level, and an associated loss in power. Koenig et al. (2008) showed how the conditional error principle of Müller and Schäfer (2001) may be used to extend the Dunnett test (Dunnett, 1955) to a two‐stage design with flexible treatment selection. This has been shown to compare well in terms of power with competing methods (Friede & Stallard, 2008).

There is a smaller body of work on clinical trials with subgroup selection, which has mainly used the combination testing approach, although methods based on the conditional error principle (Friede et al., 2012; Placzek & Friede, 2019; Stallard, Hamborg, Parsons, & Friede, 2014) and the group‐sequential approach (Magnusson & Turnbull, 2013) have also been proposed. The number of subgroups considered is usually small and different assumptions are made regarding the subgroup structure. For instance, Placzek and Friede (2019) consider nested subgroups which might arise from using different thresholds on a continuous (or at least ordinal) biomarker. An overview is provided in Ondra et al. (2016).

Although the development of some of the methods was motivated by applications in later development phases, that is, the seamless progression from phase II to phase III, the methods are more widely applicable of course. The focus on later development phases arises mainly because type I error rate is often not considered an issue in early development phases. However, in development programmes that are different from traditional ones in that they do not include at least two independent confirmatory trials, this might be different as then evidence from earlier trials will also be considered by regulators for replication of the effect demonstrated in a single phase III trial. These situations typically arise with rare diseases, in particular, rare cancers where there is a move toward platform trials including basket and umbrella trials (Woodcock & LaVange, 2017). The application of the simulation model and its implementation in the R package asd are subject to future research.

As the title of the paper by Woodcock and LaVange (2017) indicates, sometimes the application of both treatment and population selection might be of interest, in particular, in early clinical development. Motivated by late stage confirmatory trials, here we considered only designs with either treatment or subgroup selection, but the testing procedure, simulation model, and the implementation could be extended to designs where both treatments and subgroups are selected. Again, this could be subject to further research.

Throughout the manuscript, we assume that the hypothesis tests are based on a single primary outcome and that this is not changed during the trial. We consider this the likely scenario for confirmatory trials, although the adaptation of endpoints has been discussed in the literature (Kieser, 2005). However, additional information arising from other outcomes can be used to support the interim decisions. As discussed by Chataway et al. (2011), these need not be surrogate endpoints.

The testing procedures, the simulation model, and their implementation in the R package asd are quite general and are applicable to a variety of outcomes including time‐to‐event endpoints. However, the assumptions made including the *p‐clud* condition must be satisfied at least asymptotically for the testing procedure and the simulation model to be valid. For time‐to‐event endpoints, these can be easily violated resulting in substantial inflation of the type I error rate (Bauer & Posch, 2004). The main reason lies in the fact that patients with censored observations in one data look will contribute data to the next look, which is not an issue as long as the information is restricted to the censored observation times and does not include other information such as baseline characteristics or other outcomes. This complication motivated some authors including Wassmer (2006) and Jahn‐Eimermacher and Ingel (2009) to propose extensions of the combination test approach which deal with the specific issues of time‐to‐event endpoints. An overview and a discussion is provided in Chapter 9 of Wassmer and Brannath (2015).

There are of course some further limitations. Here, we focused very much on hypothesis testing, although the estimation of the treatment effects is equally important. There has been some interest in improved estimators in adaptive seamless designs with treatment (Bowden & Glimm, 2014; Brannath et al., 2009; Posch et al., 2005; Stallard & Kimani, 2018) or subgroup selection (Kimani et al., 2015, 2018), in particular, in more recent years.

With regard to interim decisions, we considered here only fairly straightforward rules although Bayesian statistics (e.g., predictive probabilities (Brannath et al., 2009)) are also used as the basis for interim decisions. Currently, we consider expanding the R package asd in this direction.

## CONFLICT OF INTEREST

The authors have declared no conflict of interest.

### Open Research Badges

This article has earned an Open Data badge for making publicly available the digitally‐shareable data necessary to reproduce the reported results. The data is available in the Supporting Information section.

This article has earned an open data badge “**Reproducible Research**” for making publicly available the code necessary to reproduce the reported results. The results reported in this article could fully be reproduced.

## Supporting information

Supporting Information

## DERIVATION OF DATA SIMULATION MODELS

We consider first treatment selection designs. Let ${\hat{\mu }}_{kj}^{\left(i\right)}$ be the estimate for treatment *k*, k=0,1,…,K with k=0 corresponding to control, at stage *j* , j=1,…,J, for endpoint *i*, i=1,2.

Assume, as often holds at least asymptotically, that ${\hat{\mu }}_{kj}^{\left(i\right)}$ is normally distributed with

$${\hat{\mu }}_{kj}^{\left(i\right)}∼N\left(\mu_{k}^{\left(i\right)},{\left(I_{kj}^{\left(i\right)}\right)}^{-1}\right),$$

with

$$cov\left({\hat{\mu }}_{kj}^{\left(i\right)},{\hat{\mu }}_{k^{\prime }j^{\prime }}^{\left(i\right)}\right)=0,k\neq k^{\prime },$$

$$cov\left({\hat{\mu }}_{kj}^{\left(i\right)},{\hat{\mu }}_{kj^{\prime }}^{\left(i\right)}\right)={\left(I_{k\max\{j,j^{\prime }\}}^{\left(i\right)}\right)}^{-1}$$

and

$$cov\left({\hat{\mu }}_{kj}^{\left(i\right)},{\hat{\mu }}_{kj^{\prime }}^{\left(i^{\prime }\right)}\right)=\rho \sqrt{{\left(I_{k\max\{j,j^{\prime }\}}^{\left(i\right)}\right)}^{-1}{\left(I_{k\max\{j,j^{\prime }\}}^{\left(i^{\prime }\right)}\right)}^{-1}},i\neq i^{\prime }.$$

For independent normal random variables with known variance σ^2^, we have $I_{k\max\{j,j^{\prime }\}}^{\left(i\right)}=\sigma^{2}/n_{kj}^{\left(i\right)}$ where $n_{kj}^{\left(i\right)}$ is the number of observations at look *j* for treatment *k* on endpoint *i*.

Let ${\hat{\theta }}_{kj}^{\left(i\right)}={\hat{\theta }}_{kj}^{\left(i\right)}-{\hat{\theta }}_{0j}^{\left(i\right)},k=1,\ldots ,K$, $I_{kj}^{\left(i\right)}={\left({\left(I_{kj}^{\left(i\right)}\right)}^{-1}+{\left(I_{0j}^{\left(i\right)}\right)}^{-1}\right)}^{-1}$ and $S_{kj}^{\left(i\right)}={\hat{\theta }}_{kj}^{\left(i\right)}I_{kj}^{\left(i\right)}$.

Let $\lambda_{kj}^{\left(i\right)}={\left(I_{kj}^{\left(i\right)}\right)}^{-1}/{\left(I_{0j}^{\left(i\right)}\right)}^{-1}$. If $\lambda_{kj}^{\left(i\right)}$ is constant, say equal to λ, and $I_{kj}^{\left(i\right)}=I_{k}^{\left(i\right)}$ for all *k* (for a single sample of known‐variance normals, this is equivalent to assuming that sample sizes are the same for all experimental treatments). In this case, considering first a single endpoint, we get

(A1)
$$\left(\begin{array}{c} S_{11}^{\left(1\right)}\\ \vdots \\ S_{K1}^{\left(1\right)}\\ \vdots \\ S_{1J}^{\left(1\right)}\\ \vdots \\ S_{KJ}^{\left(1\right)} \end{array}\right)∼N\left(\left(\begin{array}{c} \theta_{1}^{\left(1\right)}I_{1}^{\left(1\right)}\\ \vdots \\ \theta_{K}^{\left(1\right)}I_{1}^{\left(1\right)}\\ \vdots \\ \theta_{1}^{\left(1\right)}I_{J}^{\left(1\right)}\\ \vdots \\ \theta_{K}^{\left(1\right)}I_{J}^{\left(1\right)} \end{array}\right),\Sigma \right)$$

where the variance matrix is given by

$$\Sigma =\left(\begin{array}{cccc} \begin{array}{cccc} I_{1}^{\left(1\right)} & \frac{I_{1}^{\left(1\right)}}{1+\lambda } & \cdots & \frac{I_{1}^{\left(1\right)}}{1+\lambda }\\ \frac{I_{1}^{\left(1\right)}}{1+\lambda } & I_{1}^{\left(1\right)} & \ddots & \vdots \\ \vdots & \ddots & \ddots & \frac{I_{1}^{\left(1\right)}}{1+\lambda }\\ \frac{I_{1}^{\left(1\right)}}{1+\lambda } & \cdots & \frac{I_{1}^{\left(1\right)}}{1+\lambda } & I_{1}^{\left(1\right)} \end{array} & \begin{array}{cccc} I_{1}^{\left(1\right)} & \frac{I_{1}^{\left(1\right)}}{1+\lambda } & \cdots & \frac{I_{1}^{\left(1\right)}}{1+\lambda }\\ \frac{I_{1}^{\left(1\right)}}{1+\lambda } & I_{1}^{\left(1\right)} & \ddots & \vdots \\ \vdots & \ddots & \ddots & \frac{I_{1}^{\left(1\right)}}{1+\lambda }\\ \frac{I_{1}^{\left(1\right)}}{1+\lambda } & \cdots & \frac{I_{1}^{\left(1\right)}}{1+\lambda } & I_{1}^{\left(1\right)} \end{array} & \cdots & \begin{array}{cccc} I_{1}^{\left(1\right)} & \frac{I_{1}^{\left(1\right)}}{1+\lambda } & \cdots & \frac{I_{1}^{\left(1\right)}}{1+\lambda }\\ \frac{I_{1}^{\left(1\right)}}{1+\lambda } & I_{1}^{\left(1\right)} & \ddots & \vdots \\ \vdots & \ddots & \ddots & \frac{I_{1}^{\left(1\right)}}{1+\lambda }\\ \frac{I_{1}^{\left(1\right)}}{1+\lambda } & \cdots & \frac{I_{1}^{\left(1\right)}}{1+\lambda } & I_{1}^{\left(1\right)} \end{array}\\ \begin{array}{cccc} I_{1}^{\left(1\right)} & \frac{I_{1}^{\left(1\right)}}{1+\lambda } & \cdots & \frac{I_{1}^{\left(1\right)}}{1+\lambda }\\ \frac{I_{1}^{\left(1\right)}}{1+\lambda } & I_{1}^{\left(1\right)} & \ddots & \vdots \\ \vdots & \ddots & \ddots & \frac{I_{1}^{\left(1\right)}}{1+\lambda }\\ \frac{I_{1}^{\left(1\right)}}{1+\lambda } & \cdots & \frac{I_{1}^{\left(1\right)}}{1+\lambda } & I_{1}^{\left(1\right)} \end{array} & \begin{array}{cccc} I_{2}^{\left(1\right)} & \frac{I_{2}^{\left(1\right)}}{1+\lambda } & \cdots & \frac{I_{2}^{\left(1\right)}}{1+\lambda }\\ \frac{I_{2}^{\left(1\right)}}{1+\lambda } & I_{2}^{\left(1\right)} & \ddots & \vdots \\ \vdots & \ddots & \ddots & \frac{I_{2}^{\left(1\right)}}{1+\lambda }\\ \frac{I_{2}^{\left(1\right)}}{1+\lambda } & \cdots & \frac{I_{2}^{\left(1\right)}}{1+\lambda } & I_{2}^{\left(1\right)} \end{array} & \cdots & \begin{array}{cccc} I_{2}^{\left(1\right)} & \frac{I_{2}^{\left(1\right)}}{1+\lambda } & \cdots & \frac{I_{2}^{\left(1\right)}}{1+\lambda }\\ \frac{I_{2}^{\left(1\right)}}{1+\lambda } & I_{2}^{\left(1\right)} & \ddots & \vdots \\ \vdots & \ddots & \ddots & \frac{I_{2}^{\left(1\right)}}{1+\lambda }\\ \frac{I_{2}^{\left(1\right)}}{1+\lambda } & \cdots & \frac{I_{2}^{\left(1\right)}}{1+\lambda } & I_{2}^{\left(1\right)} \end{array}\\ \vdots & \vdots & \ddots & \vdots \\ \begin{array}{cccc} I_{1}^{\left(1\right)} & \frac{I_{1}^{\left(1\right)}}{1+\lambda } & \cdots & \frac{I_{1}^{\left(1\right)}}{1+\lambda }\\ \frac{I_{1}^{\left(1\right)}}{1+\lambda } & I_{1}^{\left(1\right)} & \ddots & \vdots \\ \vdots & \ddots & \ddots & \frac{I_{1}^{\left(1\right)}}{1+\lambda }\\ \frac{I_{1}^{\left(1\right)}}{1+\lambda } & \cdots & \frac{I_{1}^{\left(1\right)}}{1+\lambda } & I_{1}^{\left(1\right)} \end{array} & \begin{array}{cccc} I_{2}^{\left(1\right)} & \frac{I_{2}^{\left(1\right)}}{1+\lambda } & \cdots & \frac{I_{2}^{\left(1\right)}}{1+\lambda }\\ \frac{I_{2}^{\left(1\right)}}{1+\lambda } & I_{2}^{\left(1\right)} & \ddots & \vdots \\ \vdots & \ddots & \ddots & \frac{I_{2}^{\left(1\right)}}{1+\lambda }\\ \frac{I_{2}^{\left(1\right)}}{1+\lambda } & \cdots & \frac{I_{2}^{\left(1\right)}}{1+\lambda } & I_{2}^{\left(1\right)} \end{array} & \cdots & \begin{array}{cccc} I_{J}^{\left(1\right)} & \frac{I_{J}^{\left(1\right)}}{1+\lambda } & \cdots & \frac{I_{J}^{\left(1\right)}}{1+\lambda }\\ \frac{I_{J}^{\left(1\right)}}{1+\lambda } & I_{J}^{\left(1\right)} & \ddots & \vdots \\ \vdots & \ddots & \ddots & \frac{I_{J}^{\left(1\right)}}{1+\lambda }\\ \frac{I_{J}^{\left(1\right)}}{1+\lambda } & \cdots & \frac{I_{J}^{\left(1\right)}}{1+\lambda } & I_{J}^{\left(1\right)} \end{array} \end{array}\right).$$

Note that we can rewrite the right hand side of (A1) as

$$N\left(I^{\left(1\right)}⊗\theta^{\left(1\right)},\Sigma^{\left(GS\right)}\left(I^{\left(1\right)}\right)⊗\Sigma_{K}^{\left(CS\right)}\left(\frac{1}{1+\lambda }\right)\right)$$

where *I* ^(1)^ and θ^(1)^ denote, respectively, the vectors ${\left(I_{1}^{\left(1\right)},\ldots ,I_{J}^{\left(1\right)}\right)}^{\prime }$, and ${\left(\theta_{1}^{\left(1\right)},\ldots ,\theta_{K}^{\left(1\right)}\right)}^{\prime }$ and

$$\Sigma_{K}^{\left(CS\right)}\left(r\right)=\left(\begin{array}{cccc} 1 & r & \cdots & r\\ r & 1 & \ddots & \vdots \\ \vdots & \ddots & \ddots & r\\ r & \cdots & r & 1 \end{array}\right)$$

and

$$\Sigma^{\left(GS\right)}\left(I^{\left(1\right)}\right)=\left(\begin{array}{cccc} I_{1}^{\left(1\right)} & I_{1}^{\left(1\right)} & \cdots & I_{1}^{\left(1\right)}\\ I_{1}^{\left(1\right)} & I_{2}^{\left(1\right)} & \cdots & I_{2}^{\left(1\right)}\\ \vdots & \vdots & \ddots & \vdots \\ I_{1}^{\left(1\right)} & I_{2}^{\left(1\right)} & \cdots & I_{J}^{\left(1\right)} \end{array}\right)$$

denote variance matrices of the K×K complex symmetric form and of that obtained in the usual group‐sequential setting.

When both endpoints are considered, the vector

$${\left(S_{11}^{\left(1\right)},\ldots ,S_{K1}^{\left(1\right)},\ldots ,S_{1J}^{\left(1\right)},\ldots ,S_{KJ}^{\left(1\right)},S_{11}^{\left(2\right)},\ldots ,S_{K1}^{\left(2\right)},\ldots ,S_{1J}^{\left(2\right)},\ldots ,S_{KJ}^{\left(2\right)}\right)}^{\prime }$$

is normally distributed with mean

$$\left(\begin{array}{c} \theta^{\left(1\right)}⊗I^{\left(1\right)}\\ \theta^{\left(2\right)}⊗I^{\left(2\right)} \end{array}\right)$$

and variance

$$\left(\begin{array}{cc} \Sigma^{\left(GS\right)}\left(I^{\left(1\right)}\right) & \rho \Sigma^{\left(GS\right)}\left(I^{\left(12\right)}\right)\\ \rho \Sigma^{\left(GS\right)}\left(I^{\left(12\right)}\right) & \Sigma^{\left(GS\right)}\left(I^{\left(2\right)}\right) \end{array}\right)⊗\Sigma_{K}^{\left(CS\right)}\left(\frac{1}{1+\lambda }\right)$$

where is a *J*‐dimensional vector with $I_{j}^{\left(12\right)}=\sqrt{I_{j}^{\left(1\right)}I_{j}^{\left(2\right)}},j=1,\ldots ,J$.

We consider next designs with subgroup selection. In analogy to the notation used above, now let ${\hat{\theta }}_{kj}^{\left(i\right)}$ be the estimate of the treatment effect in subgroup *k*, k=1,…,K at stage *j* , j=1,…,J, for endpoint *i*, i=1,2. We will typically consider the case of K=2, with k=2 corresponding to the entire population and k=1 corresponding to a subgroup comprising a proportion τ of the entire population.

Let ${\left(I_{kj}^{\left(i\right)}\right)}^{-1}$ denote the variance of ${\hat{\theta }}_{kj}^{\left(i\right)}$ and let $S_{kj}^{\left(i\right)}={\hat{\theta }}_{kj}^{\left(i\right)}I_{kj}^{\left(i\right)}$. As above, we will assume that the ${\hat{\theta }}_{kj}^{\left(i\right)}$ are normally distributed with mean $\theta_{k}^{\left(i\right)}$ and variance ${\left(I_{kj}^{\left(i\right)}\right)}^{-1}$ with

$$cov\left({\hat{\theta }}_{kj}^{\left(i\right)},{\hat{\theta }}_{k^{\prime }j^{\prime }}^{\left(i\right)}\right)={\left(I_{\max\left\{k,k^{\prime }\right\}\max\left\{j,j^{\prime }\right\}}^{\left(i\right)}\right)}^{-1}$$

and

$$cov\left({\hat{\theta }}_{kj}^{\left(i^{\prime }\right)},{\hat{\theta }}_{k^{\prime }j^{\prime }}^{\left(i\right)}\right)=\rho {\left(I_{\max\left\{k,k^{\prime }\right\}\max\left\{j,j^{\prime }\right\}}^{\left(i\right)}I_{\max\left\{k,k^{\prime }\right\}\max\left\{j,j^{\prime }\right\}}^{\left(i^{\prime }\right)}\right)}^{-1/2},i\neq i^{\prime }.$$

Denote $I_{j2}^{\left(i\right)}$ by $I_{j}^{\left(i\right)}$ and assume further that $I_{j1}^{\left(i\right)}=\tau I_{j}^{\left(i\right)}$ for i=1,2,j=1,…,J (see Spiessens and Debois), then

$${\left(S_{11}^{\left(1\right)},S_{21}^{\left(1\right)},\ldots ,S_{1J}^{\left(1\right)},S_{2J}^{\left(1\right)},S_{11}^{\left(2\right)},S_{21}^{\left(2\right)},\ldots ,S_{1J}^{\left(2\right)},S_{2J}^{\left(2\right)}\right)}^{\prime }$$

is normally distributed with mean

$$\left(\begin{array}{c} \theta^{\left(1\right)}⊗I^{\left(1\right)}\\ \theta^{\left(2\right)}⊗I^{\left(2\right)} \end{array}\right)$$

and variance

$$\left(\begin{array}{cc} \Sigma^{\left(GS\right)}\left(I^{\left(1\right)}\right) & \rho \Sigma^{\left(GS\right)}\left(I^{\left(12\right)}\right)\\ \rho \Sigma^{\left(GS\right)}\left(I^{\left(12\right)}\right) & \Sigma^{\left(GS\right)}\left(I^{\left(2\right)}\right) \end{array}\right)⊗\Sigma^{\left(GS\right)}\left(\begin{array}{c} \tau \\ 1 \end{array}\right)$$

where $I^{\left(i\right)}={\left(I_{1}^{\left(i\right)},\ldots ,I_{J}^{\left(i\right)}\right)}^{\prime }$, $I^{12}={\left(I_{1}^{\left(12\right)},\ldots ,I_{J}^{\left(12\right)}\right)}^{\prime }$ with $I_{j}^{\left(12\right)}=\sqrt{I_{j}^{\left(1\right)}I_{j}^{\left(2\right)}}$, $\theta^{\left(i\right)}=\left(\theta_{1}^{\left(i\right)},\theta_{2}^{\left(i\right)}\right)$, and $\Sigma^{\left(GS\right)}\left(I\right)$ and $\Sigma_{K}^{\left(CS\right)}\left(r\right)$ are as defined above.

If more than two subgroups are considered, or if these are nested in some other way than the second being a subset of the first, the last matrix can be modified accordingly to reflect this.
